# Assessment of medical students’ leadership traits in a problem/case-based learning program

**DOI:** 10.1080/10872981.2018.1542923

**Published:** 2018-11-08

**Authors:** Samara B. Ginzburg, Jessica Schwartz, Rachel Gerber, Susan Deutsch, David E. Elkowitz, Christina Ventura-Dipersia, Youn Seon Lim, Robert Lucito

**Affiliations:** a Department of Science Education, Zucker School of Medicine at Hofstra/Northwell, Hempstead, NY, USA; b Zucker School of Medicine at Hofstra/Northwell, Hempstead, NY, USA; c Hofstra Northwell School of Graduate Nursing and Physician Assistant Studies, Hempstead, NY, USA

**Keywords:** Leadership, problem-based learning, case-based learning, traits, teamwork, undergraduate medical education, self-directed learning

## Abstract

**Background**: Healthcare delivery is shifting to team-based care and physicians are increasingly relied upon to lead and participate in healthcare teams. Educational programs to foster the development of leadership qualities in medical students are needed to prepare future physicians for these roles.

**Objective**: Evaluate the development of leadership attributes in medical students during their first 2 years of medical school while participating in leadership training integrated into a problem/case-based learning program utilizing the Leadership Traits Questionnaire assessment tool.

**Design**: Ninety-eight students enrolled at Zucker School of Medicine participated in Patient-Centered Explorations in Active Reasoning, Learning and Synthesis (PEARLS), a hybrid problem/case-based learning program, during the first and second years of medical school. The Leadership Traits Questionnaire, designed to measure 14 distinct leadership traits, was utilized. It was administered to students, peers in students’ PEARLS groups and their faculty facilitators. Participants completed questionnaires at three-time points during the study. Likert scale data obtained from the questionnaire was analyzed using a two-level Hierarchal Linear Model.

**Results**: Complete data sets were available for 84 students. Four traits, including self-assured, persistent, determined, and outgoing, significantly increased over time by measurements of both peer and facilitator-rated assessments. Six additional traits significantly increased over time by measurement of facilitator-rated assessment. By contrast, a majority of student self-rated assessments trended downward during the study.

**Conclusions**: Medical students demonstrated development of several important leadership traits during the first 2 years of medical school. This was accomplished while participating in the PEARLS program and without the addition of curricular time. Future work will examine the impact of third year clerkships on leadership traits.

## Introduction

The provision of healthcare in the United States is dynamically evolving in response to a complex set of financial and societal pressures from patients, providers, organizations, and policy-makers [1,2]. As a result, the current structure of healthcare relies increasingly on collaborative models, and physicians must be able to lead and work in teams to provide high-quality, cost-effective patient care [3–5]. Medical schools must set the foundation for this work by providing their students opportunities to develop and enhance leadership skills.

Recent literature pertaining to leadership training in undergraduate medical education (UME) offers various paradigms for the structure and content of leadership curricula or programs [1,2]. Of the medical schools with leadership development programs, some evaluate student satisfaction with their learning [6–8], while others examine students’ confidence in their leadership abilities or teaching skills after participating in leadership programs [9–12]. Several studies measure students’ preparedness or willingness to lead in specific scenarios, such as community service [13–17] or quality improvement [18]. There have also been reports of increases in understanding of leadership as part of a physician’s professional responsibility among students participating in leadership programs [19,20]. The majority of studies assessing development of leadership in UME utilize a measurement of students’ self-assessments or self-reporting [6–12], while a few report faculty supervisors’ assessments of leadership development among students [16,21], but to our knowledge, none include assessments by peers. In addition, the majority of tools employed to date to assess leadership development utilize situation-based questions or narrative comments rather than measuring discrete leadership traits [15–17,20].

We previously showed utilizing an internally developed tool that integration of a longitudinal curriculum in leadership development into our hybrid problem-based/case-based learning (PBL/CBL) program, Patient-Centered Explorations in Active Reasoning, Learning and Synthesis (PEARLS) is an effective way to develop leadership qualities in our medical students [21]. To both deepen and better characterize the impact of this leadership training, we sought to include perspectives from three different evaluator groups who participate in this program – students, peers, and faculty facilitators. In addition, we were interested in quantifying specific leadership traits impacted by this program. Accordingly, we utilized the Leadership Traits Questionnaire (LTQ) [22], developed by Peter Northouse, an expert in the field of leadership research, to evaluate leadership development in medical students participating in PEARLS. The LTQ measures 14 leadership traits and reliably assesses an individual’s traits when completed by multiple people familiar with the person being assessed [22]. To our knowledge, it has not previously been utilized in the UME setting. We hypothesized that participation in our PEARLS program, which includes leadership training, would nurture development of leadership traits in our medical students when evaluated from the perspectives of students, peers, and faculty facilitators.

## Methods

The present study took place during academic years 2015–2016 (year 1) and 2016–2017 (year 2). Students participated in the Zucker School of Medicine PEARLS program, which includes leadership development as previously described [21,23]. The core elements of the leadership curriculum for students included serving as the ‘leader’ for PEARLS sessions on a rotating basis (setting the agenda for the group, managing group dynamics, and encouraging participation from group members), developing and presenting targeted learning exercises (‘triggers’) during each session and participating in wrap-up discussions related to leadership [21]. Each student was assigned to a PEARLS group of seven or eight peers with a faculty facilitator. Facilitators were responsible for guiding the PEARLS process and not for delivery of content. The PEARLS program includes six courses, which range in length from 6 to 12 weeks. Student groups and facilitators changed at the end of each course. A single cohort of 98 students was enrolled in the study (Table 1).

**Table 1.** Student and facilitator demographic characteristics.

Demographic characteristics				
Student demographics (*N* = 101)^a^			Freq	%
Gender		Male	51	49.5
		Female	50	50.5
Race		African American	8	7.9
		Asian	16	15.8
		White	57	56.4
		Mixed races	7	6.9
		No responses	13	12.9
			Mean	SD
	Age (Year)^b^		23.76	1.95
**Facilitator demographics**				
***Course 1* (*N* = 12)**			Freq	%
Gender		Male	7	58
		Female	5	42
Degree		MD	9	75
		DO	1	8
		PhD	2	17
Courses facilitated			Mean	3.5
***Course 3* (*N* = 11)**			Freq	%
Gender		Male	8	73
		Female	3	27
Degree		MD	6	55
		DO	1	9
		PhD	4	36
Courses Facilitated			Mean	3.9
***Course 6* (*N* = 12)**			Freq	%
Gender		Male	6	50
		Female	6	50
Degree		MD	5	42
		DO	3	25
		PhD	4	33
Courses Facilitated			Mean	3.7

Note. ^a^Total in student demographics does not represent sample size for full analyses due to missing values. For self-assessment, missing values were 6, 6, 7; for peer-assessment, 6, 0, 3; for facilitator-assessment, 2, 0, 3 for Courses 1, 3, and 6, respectively. ^b^Age is calculated between birthday and 8 January 2015 and 2016.

### Leadership Traits Questionnaire

The LTQ was used in its original format with the 5-point Likert scale for the 14 traits (Table 2) in year 1 of this study [22]. Statistical analysis performed at the end of year 1 showed similar responses to all questions. To differentiate the responses of the study participants, a 10-point Likert scale was applied to the 14 traits in year 2 of the study. Measures from year 2 underwent a linear transformation from a 1–10 scale back to the original 1–5 scale in order to compare them with measures from year 1 [24].

Student participation in this study was voluntary. Students were asked to complete an LTQ on themselves and all other student members of their PEARLS group at three-time points: the end of Course 1 (a 6-week course) and the midpoint (week 6) of Courses 3 and 6, about half way through years 1 and 2, respectively. Facilitators (Table 1) also completed the LTQ for all students assigned to their groups at the same three-time points.

The LTQ survey was generated using Qualtrics (QUALTRICS, Provo, UT, USA) at the first time point and Baseline (Campus Labs, Buffalo, NY, USA) at the second and third time points. Surveys were distributed through email and were accessible via computer or mobile platforms. Students were provided time to complete surveys during the end of a designated PEARLS session at the three-time points. Facilitators left the rooms while students completed their surveys. Facilitators received instructions to complete their LTQ surveys within 1 week of receipt.

## Analysis

For both facilitator and student surveys, all results were de-identified before any calculations or analysis of the data took place. All self, peer, and facilitator completed LTQ forms with greater than 90% of the questions answered were included in the analysis [25]. For peer-completed surveys, the average peer response per trait was calculated. All students, whose average peer response had a Cronbach’s alpha value >0.8 for all 14 traits, were included in the analysis. With the balance, data missing at random was imputed using the EM missing values procedure. Inspection of means and standard deviations indicated that the EM procedure caused only minimal difference to the data (*i.e*., differences noticeable only at the .01 level). As a result, the total number of participants is shown in Table 1.

A two-level Hierarchal Linear Model (HLM) was used to evaluate leadership growth in PEARLS (Appendix 2, 3, 4). HLM analysis was chosen over traditional regression analysis because the use of repeated measures was likely to generate correlated errors across time. HLM analysis was chosen over structural equation modeling because HLM is a more robust model and can sufficiently model datasets with missing data. At level 1 of the model, time was used as a predictor of leadership outcomes of the within-subject variable. The slope and intercept parameters of the equation for leadership were analyzed for evidence of random effects. Evidence of random effects indicates significant inter-individual variability in the parameters and is required before testing between-participants variables as level 2 predictors. At level 2 of the model, separate equations were created for the intercept and slope parameters of the level 1 equation. To compare the initial leadership scores, a one-way analysis of variance (ANOVA) was used. *Post hoc* tests were conducted using the Bonferroni correction.

## Results

**Figure 1.**
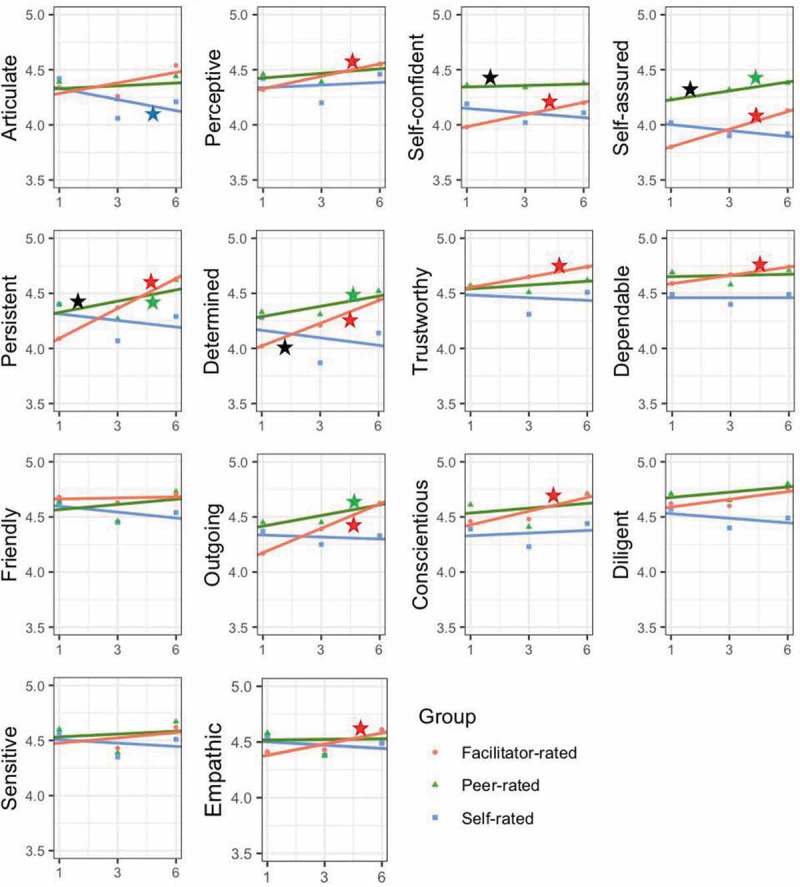
Fixed effect regression model for each leadership trait: the *Y*-axis is the average 5-point Likert scale rating for each student as determined by self, facilitator, and peer evaluator groups. The *X*-axis is the course number in the curriculum. Notes. *p* Value <.05 in comparison of intercepts; *p* Value <.05 in facilitator-rated measure trend over time; *p* Value <.05 in peer-rated measure trend over time; *p* Value <.05 in self-rated measure trend over time.

Key findings regarding the development of particular traits as well as overall trends in student leadership trait development for the cohort of students followed longitudinally are shown in Figure 1. Of the 14 traits examined in this study, none were found to increase across all three evaluator groups. There were four traits that significantly improved (alpha level = 0.05) as measured by two evaluator groups, facilitator and peer, and included *self-assured, persistent, determined*, and *outgoing*. There were six traits that significantly improved as measured by one evaluator group, facilitator, and included *perceptive, self-confident, trustworthy, dependable, conscientious*, *and empathic*.

The general trend of student self-rated assessments, however, was to decrease over time (only *articulate* was statistically significant). There were no statistically significant differences of initial leadership measures between groups as determined by one-way ANOVA except *self-confident, persistent*, and *determined*. The *post hoc* tests revealed that facilitators’ evaluations were statistically significantly lower than the other evaluator groups at baseline (Appendix 1).

## Discussion

Moving forward, physicians will be expected to actively shape and support interprofessional, team-based models of healthcare delivery. Medical schools have begun preparing graduates for this role via inclusion of leadership training during UME. Our PEARLS program promotes higher order discussion and critical thinking skills important for successful leaders to possess, and, in doing so, creates an environment conducive to leadership trait development. In the present study, we demonstrated the ability to positively impact medical students’ development of several key leadership traits through student participation in our PEARLS program (Table 3).

**Table 2.** Leadership traits on Leadership Trait Questionnaire.

Leadership Traits on LTQ [22]
Articulate
Perceptive
Self-confident
Self-assured
Persistent
Determined
Trustworthy
Dependable
Friendly
Outgoing
Conscientious
Diligent
Sensitive
Empathic

**Table 3.** Leadership traits significantly improved from baseline.

Peer and facilitator evaluator groups	Facilitator evaluator group	Student self-evaluator group
Self-assured	Perceptive	Articulate^a^
Persistent	Self-confident	
Determined	Trustworthy	
Outgoing	Dependable	
	Conscientious Empathic	

^a^Denotes statistically significant decrease.

Surprisingly, student self-rated assessments were very different from peer- and facilitator-rated assessments in aggregate. Self-assessments trended toward a slight decrease or no change in leadership traits over time, with *articulate* significantly decreasing during the study. The only two traits on self-assessment that trended toward increasing over time were *conscientious* and *perceptive*. Interestingly, these two traits are more reflective or introspective as opposed to those that are outwardly manifested. Assessing one’s own progress is a challenging exercise. The reliability of medical students’ self-assessments has been questioned previously [26]. It has been shown that the accuracy of medical students’ self-assessments depends upon external sources of feedback to help calibrate them [27]. In our study, students’ baseline self-assessments were gathered prior to receiving formal feedback from their facilitators. By time points two and three, students had received feedback from their facilitators and had also assessed their peers, both of which may have contributed to students’ rating themselves more conservatively at these later points during the study. Another possible factor affecting students’ self-assessments is the ‘impostor syndrome’, a phenomenon characterized by self-doubt and fear of being discovered as an intellectual fraud [28]. When analyzing these outcomes, we must consider that PEARLS facilitators have experience observing a diverse range of students in this educational setting and, as a result, may be more accurate in their assessments. Additionally, assessments made by the peer evaluator group represent aggregate data collected from all peers assessing a given student. These compiled values may be more representative of a student’s performance than a single student’s self-assessment of his or her leadership traits. The close alignment of assessments made by the peer and facilitator evaluator groups further suggests that there was notable development of leadership traits amongst this cohort for those traits that statistically significantly increased during this study.

The beneficial impact of the program on leadership development in medical students is underscored by the overall alignment between the peer and facilitator assessments for the majority of leadership traits. This result is among the first to demonstrate development of discrete leadership traits not limited to a specific scenario or experience in medical students as the result of curricular engagement. Additionally, in contrast to previous research, which suggests that only leadership skills are amenable to change through experience, our work shows demonstrable change in leadership traits [29–32]. Furthermore, it is important to note that all these developments took place without the addition of curricular time devoted solely to leadership education.

Our study had several limitations. We analyzed results from one cohort of 98 students from our institution and recognize that results obtained from this student sample may have been different had additional cohorts been studied – either from within or beyond our own institution. In addition, we were not able to utilize a control group, as all students in the cohort were enrolled in our PBL/CBL program. As the nature of the curriculum prevents facilitators from making their observations of students blindly, we acknowledge that their assessments of students’ leadership traits may have been affected by subjective factors including improved perception and interpersonal bonds formed over time, as in the halo effect [33]. While the LTQ is a useful tool for measuring general leadership traits, it is also possible that some of the traits included on the form are not equally necessary for medical students or physicians and further analysis of this tool to determine its appropriateness for the medical student population is warranted.

In future work, we will study multiple cohorts of students and follow them through the third and fourth years of medical school. During that time, we will examine if students’ self-assessment of their leadership traits become more aligned with those of peer and facilitator evaluator groups in the setting of receiving additional external feedback at multiple points in time. In addition, we are interested in examining whether clerkship experiences further impact leadership trait development.

## Appendices

**Appendix 1:** Comparison of fixed effect of initial leadership trait scores

Leadership	Self-rated		Peer-rated		Facilitator-rated				
Trait	*β*	SE	*β*	SE	*β*	df	*F*	*η*^2^ (%)	SE
Articulate	4.43	0.10	4.24	0.07	4.19	0.11	2,2267	1.78	0.16
Perceptive	4.31	0.10	4.33	0.05	4.22	0.11	2,2267	0.42	0.04
Self-confident	4.19	0.11	4.19	0.07	3.87	0.13	2,2267	3.02	0.27
Self-assured	**4.05^A^**	0.13	**4.07^B^**	0.07	**3.64^AB^**	0.13	2,2267	**4.57***	0.40
Persistent	**4.37^A^**	0.12	**4.22^B^**	0.05	**3.82^AB^**	0.11	2,2267	**8.36***	0.73
Determined	**4.23^A^**	0.12	**4.20^B^**	0.07	**3.82^AB^**	0.12	2,2267	**4.65***	0.41
Trustworthy	4.51	0.09	4.42	0.06	4.46	0.09	2,2267	0.31	0.03
Dependable	4.46	1.00	4.45	0.06	4.51	0.08	2,2267	0.00	0.00
Friendly	4.65	0.09	4.44	0.06	4.65	0.09	2,2267	2.23	0.20
Outgoing	**4.35^A^**	0.10	4.26	0.07	**3.95^A^**	0.12	2,2267	**4.51***	0.40
Conscientious	4.30	0.11	4.43	0.05	4.30	0.10	2,2267	0.69	0.06
Diligent	4.57	0.10	4.58	0.04	4.52	0.09	2,2267	0.16	0.01
Sensitive	4.53	0.10	4.35	0.07	4.43	0.11	2,2267	0.90	0.08
Empathic	4.53	0.09	4.36	0.07	4.28	0.12	2,2267	1.78	0.16

Notes. **p* < .05.

^A^*Post hoc* test self-rated vs. facilitator-rated *p* < .05.

^B^*Post hoc* test peer-rated vs. facilitator-rated *p* < .05.

## Appendix

**Appendix 2.** Hierarchical linear model for self-rated traits of leadership

	Fixed effects				Random effects					
	Baseline		Trend		Level 1		Baseline		Trend	
Leadership trait	→_00_	SE	→_10_	SE	Var	SD	♥_0i_	SD	♥_1i_	SD
Articulate	**4.43***	0.10	**−0.10***	0.42	0.27	0.52	**0.16***	0.39	0.01	0.13
Perceptive	**4.31***	0.10	0.02	0.04	0.31	0.55	**0.11***	0.33	0.00	0.00
Self-confident	**4.19***	0.11	−0.42	0.40	0.27	0.52	**0.44***	0.66	0.00	0.00
Self-assured	**4.05***	0.13	−0.05	0.05	0.45	0.67	**0.36***	0.60	0.00	0.00
Persistent	**4.37***	0.12	−0.06	0.05	0.41	0.64	0.03	0.18	0.00	0.00
Determined	**4.23***	0.12	−0.07	0.05	0.41	0.64	**0.28***	0.53	0.00	0.00
Trustworthy	**4.51***	0.09	−0.02	0.04	0.26	0.51	0.12	0.34	0.00	0.00
Dependable	**4.46***	1.00	0.00	0.04	0.26	0.05	**0.18***	0.42	0.01	0.03
Friendly	**4.65***	0.09	−0.05	0.04	0.25	0.50	**0.14***	0.37	0.00	0.06
Outgoing	**4.35***	0.10	−0.2	0.04	0.30	0.55	**0.17***	0.41	0.02	0.14
Conscientious	**4.30***	0.11	0.02	0.05	0.35	0.59	**0.14***	0.37	0.01	0.59
Diligent	**4.57***	0.10	−0.04	0.04	0.26	0.52	**0.16***	0.39	0.00	0.05
Sensitive	**4.53***	0.10	−0.03	0.04	0.28	0.52	**0.15***	0.39	0.28	0.53
Empathic	4.53	0.09	−0.03	0.04	0.23	0.48	**0.22***	0.47	0.00	0.00

**p* < .05.

## Appendix

**Appendix 3.** Hierarchical linear model for peer-rated traits of leadership

	Fixed effects				Random effects					
	Initial		Trend		Level 1		Baseline		Trend	
Leadership trait	→_00_	SE	→_10_	SE	Var	SD	♥_0i_	SD	♥_1i_	SD
Articulate	**4.24***	0.07	0.05	0.03	0.14	0.37	0.06	0.21	0.00	0.05
Perceptive	**4.33***	0.05	0.04	0.03	0.10	0.31	0.00	0.00	**0.01***	0.09
Self-confident	4.19	0.07	0.05	0.03	0.12	0.35	**0.09***	0.30	0.00	0.07
Self-assured	**4.07***	0.07	**0.09***	0.03	0.13	0.36	**0.08***	0.28	0.00	0.06
Persistent	**4.22***	0.05	**0.09***	0.03	0.10	0.32	0.00	0.00	0.00	0.06
Determined	**4.20***	0.07	**0.07***	0.04	0.16	0.39	0.01	0.09	0.10	0.10
Trustworthy	4.42	0.06	0.02	0.03	0.09	0.31	0.02	0.13	0.01	0.08
Dependable	**4.45***	0.06	0.04	0.03	0.10	0.32	0.00	0.00	**0.01***	0.10
Friendly	**4.44***	0.06	0.03	0.26	0.09	0.31	**0.05***	0.21	0.00	0.00
Outgoing	**4.26***	0.07	**0.08***	0.03	0.12	0.34	0.07	0.27	0.00	0.00
Conscientious	**4.43***	0.05	0.03	0.03	0.08	0.28	0.00	0.02	**0.01***	0.08
Diligent	**4.58***	0.04	0.02	0.02	0.06	0.25	0.00	0.00	**0.01***	0.08
Sensitive	**4.35***	0.07	0.05	0.03	0.14	0.38	**0.04***	0.19	0.00	0.00
Empathic	**4.36***	0.07	0.04	0.03	0.13	0.06	**0.05***	0.22	0.00	0.00

**p* < .05.

## Appendix

**Appendix 4.** Hierarchical linear model for facilitator-rated traits of leadership

	Fixed effects				Random effects					
	Baseline		Trend		Level 1		Baseline		Trend	
Leadership trait	→_00_	SE	→_10_	SE	Var	SD	♥_0i_	SD	♥_1i_	SD
Articulate	**4.19***	0.11	0.10	0.05	0.43	0.65	**0.09***	0.29	0.00	0.00
Perceptive	**4.22***	0.11	**0.11***	0.05	0.42	0.64	0.03	0.18	0.00	0.00
Self-confident	**3.87***	0.13	**0.11***	0.05	0.47	0.68	0.27	0.51	0.00	0.00
Self-assured	**3.64***	0.13	**0.16***	0.05	0.47	0.68	**0.21***	0.46	0.00	0.00
Persistent	**3.82***	0.11	**0.27***	0.05	0.44	0.66	0.04	0.19	0.00	0.00
Determined	**3.82***	0.12	**0.21***	0.05	0.44	0.66	**0.21***	0.45	0.00	0.00
Trustworthy	**4.46***	0.09	**0.09***	0.04	0.29	0.54	0.03	0.18	0.00	0.06
Dependable	**4.51***	0.08	**0.08***	0.04	0.24	0.49	0.00	0.00	0.01	0.10
Friendly	**4.65***	0.09	0.01	0.04	0.29	0.55	0.00	0.00	0.01	0.07
Outgoing	**3.95***	0.12	**0.22***	0.05	0.41	0.64	**0.18***	0.42	0.00	0.00
Conscientious	**4.30***	0.10	**0.12***	0.04	0.32	0.56	0.53	0.23	0.00	0.07
Diligent	**4.52***	0.09	0.07	0.04	0.27	0.52	0.03	0.16	0.01	0.08
Sensitive	**4.43***	0.11	0.05	0.05	0.44	0.67	0.00	0.00	0.00	0.06
Empathic	**4.28***	0.12	**0.10***	0.05	0.47	0.68	0.12	0.12	0.00	0.00

**p* < .05.

## References

[1] Webb AM, Tsipis NE, McClellan TR, et al A first step toward understanding best practices in leadership training in undergraduate medical education: a systematic review. Acad Med. 2014;89(11):1–8. 25250751 10.1097/ACM.0000000000000502

[2] Neeley SM, Clyne B, Resnick-Ault D. The state of leadership education in US medical schools: results of a national survey. Med Educ Online. 2014;22(1):1–4. 10.1080/10872981.2017.1301697 PMC5419299 28298155

[3] Schneider EC, Squires D From first to last- could the U.S. health care system become the best in the world? N Engl J Med. 2017;377(10):901–904. 28708020 10.1056/NEJMp1708704

[4] Stiefel M, Nolan K. A guide to measuring the triple aim: population health, experience of care, and per capita cost(IHI Innovation Series white paper). Cambridge (MA): Institute for Healthcare Improvement 2012 [cited 2018 71]. Available from: http://www.ihi.org/resources/Pages/IHIWhitePapers/AGuidetoMeasuringTripleAim.aspx

[5] Interprofessional Education Collaborative Expert Panel Core competencies for interprofessional collaborative practice: report of an expert panel. Washington, DC: Interprofessional Education Collaborative; 2011 [cited 2018 71] Available from: https://www.aacom.org/docs/default-source/insideome/ccrpt05-10-11.pdf?sfvrsn=77937f97_2

[6] Prywes M, Friedman M Education for leadership in health development. Acad Med. 1991;66(4):209–210. 2012652 10.1097/00001888-199104000-00009

[7] Cadieux DC, Lingard L, Kwiatkowski D, et al Challenges in translation: lessons from using business pedagogy to teach leadership in undergraduate medicine. Teach Learn Med. 2016;29(2):207–215. 27813682 10.1080/10401334.2016.1237361

[8] Ganzel T Actively engaging students in a quality improvement initiative. Med Educ. 2004;38(5):562–563. 15107111 10.1111/j.1365-2929.2004.01871.x

[9] Smith KL, Petersen DJ, Soriano R, et al Training tomorrow’s teachers today: a national medical student teaching and leadership retreat. Med Teach. 2007;29(4):328–334. 17786746 10.1080/01421590701316530

[10] Coleman MM, Blatt B, Greenberg L Preparing students to be academicians: a national student-led summer program in teaching, leadership, scholarship, and academic medical career-building. Acad Med. 2012;87(12):1734–1741. 23095923 10.1097/ACM.0b013e318271cfd6

[11] O’Connell MT, Pascoe JM Undergraduate medical education for the 21st century: leadership and teamwork. Fam Med. 2004;36(suppl):S51–S56. 14961403

[12] Dobson C, Cookson J, Allgar V, et al Leadership training in the undergraduate medical curriculum. Educ Prim Care. 2008;19(5):526–529.

[13] Warde CM, Vermilion M, Uijtdehaage S A medical student leadership course led to teamwork, advocacy and mindfulness. Fam Med. 2014;46(6):459–462. 24911302

[14] Mohan CP, Mohan A HealthSTAT: a student approach to building skills needed to serve poor communities. J Health Care Poor Underserved. 2007;18(3):523–531. 17675711 10.1353/hpu.2007.0063

[15] Goldstein AO, Calleson D, Bearman R, et al Teaching advanced leadership skills in community service (ALSCS) to medical students. Acad Med. 2009;84(6):654–764. 10.1097/ACM.0b013e3181a40660 19474554

[16] Long JA, Lee RS, Federico S, et al Developing leadership and advocacy skills in medical students through service learning. J Public Health Manag Pract. 2011;17(4):369–372. 21617415 10.1097/PHH.0b013e3182140c47

[17] Carufel-Wert DA, Younkin S, Foertsch J, et al LOCUS: immunizing medical students against the loss of professional values. Fam Med. 2007;39(5):320–325. 17476604

[18] Gonsenhauser I, Beal E, Shihadeh F, et al Development and assessment of quality improvement education for medical students at the Ohio state University Medical Center. J Healthc Qual. 2012;34(6):36–42. 23163971 10.1111/j.1945-1474.2012.00160.x

[19] Bergman D, Savage C, Wahlstrom R, et al Teaching group dynamics—do we know what we are doing? An approach to evaluation. Med Teach. 2008;30(1):55–61. 18278652 10.1080/01421590701758624

[20] Agarwal A, Anderson J, Sarfaty S, et al The value of an elective in business and leadership for medical student. J Med Pract Manage. 2015;30(4):276–280. 26223111

[21] Ginzburg SB, Deutsch S, Bellissimo J, et al Integration of leadership training into a problem/case-based learning program for first- and second-year medical students. Adv Med Educ Pract. 2018;9(1):221–226. 29670414 10.2147/AMEP.S155731 PMC5898582

[22] Northouse PG Leadership: theory and practice. Thousand Oaks (CA): Sage Publications; 2016.

[23] Ginzburg S, Brenner J, Willey J Integration: a strategy for turning knowledge into action. Med Sci Educ. 2015;25(4):533–543.

[24] De Jong T, Veenhoven R, Arends L Homogenizing responses to different survey questions on the same topic: proposal of a scale homogenization method using a reference distribution. Soc Indic Res. 2014;117:275–300. 24707072 10.1007/s11205-013-0335-6 PMC3971463

[25] Bennett DA How can I deal with missing data in my study? Aust N Z J Public Health. 2001;25(5):464–469. 11688629

[26] Ward M, Gruppen L, Regehr G Measuring self-assessment: current state of the art. Adv Health Sci Educ Theory Pract. 2002;7(1):63–80. 11912336 10.1023/a:1014585522084

[27] Mann K, Van Der Vleuten C, Eva K, et al Tensions in informed self-assessment: how the desire for feedback and reticence to collect and use it can conflict. Acad Med. 2011;86(9):1120–1127. 21785309 10.1097/ACM.0b013e318226abdd

[28] Villwock JA, Sobin LB, Koester LA, et al Impostor syndrome among American medical students: a pilot study. Int J Med Educ. 2016;7(1):364–369. 27802178 10.5116/ijme.5801.eac4 PMC5116369

[29] Houghton JD, Bonham TW, Neck CP, et al The relationship between self-leadership and personality: a comparison of hierarchical factor structures. J Manag Psychol. 2004;19(4):427–441.

[30] Arvey RD, Rotundo M, Johnson W, et al The determinants of leadership role occupancy: genetic and personality factors. Leadersh Q. 2006;17(1):1–20.

[31] Johnson AM, Vernon PA, McCarthy JM, et al Nature vs. nurture: are leaders born or made? A behavior genetic investigation of leadership style. Twin Res Hum Genet. 1998;1(04):216–223. 10.1375/136905298320566195 10100814

[32] Ruvolo CM, Peterson SA, LeBoeuf JNG Leaders are made, not born. The critical role of a developmental framework to facilitate an organizational culture of development. Consult Psychol J. 2004;56(1):10–19.

[33] Murphy KR, Jako RA, Anhalt RL Nature and consequences of halo error: a critical analysis. J Appl Psychol. 1993;78(2):218–225.

